# Toxicologic Exposures in California Emergency Departments in 2011 and Its Risk Factors

**DOI:** 10.5811/westjem.2021.3.50452

**Published:** 2021-08-22

**Authors:** Shahram Lotfipour, Connie Au, Soheil Saadat, Tim Bruckner, Parvati Singh, Bharath Chakravarthy

**Affiliations:** * University of California, Irvine, Department of Emergency Medicine, Irvine, California; † Eisenhower Health, Department of Emergency Medicine, Rancho Mirage, California; ‡ University of California, Irvine, School of Medicine, Irvine, California; § University of California, Irvine, Program in Public Health, Irvine, California

## Abstract

**Introduction:**

Toxicologic exposures (TE) are a major preventable public health issue, with most cases due to unintentional causes. Although these cases are well documented and reported via the National Poison Data System, there is little information regarding toxicologic exposure cases in the emergency department (ED). The aim of this study was to identify demographic groups at risk for potential poisoning.

**Methods:**

This was a cross-sectional study. We used data from the California State Emergency Department Database (SEDD) 2011 for statistical analysis.

**Results:**

The study included 10,124,598 ED visits in California in 2011. The prevalence of TE was 383.4 (379.6–387.3) per 100,000 visits. Toxicologic exposures were most common among patients aged <10 years (555.4, 95% confidence interval [CI]: 544.5–566.5 per 100,000 visits). Overall, TE was more common among males. White patients showed the highest prevalence of TE compared to other racial groups (P <0.001). Subpopulation analysis showed Native American female patients ages 10–19 had a noticeably higher prevalence of TE (1,464.4, 95% CI: 802.9–2444.9 per 100,000). The prevalence of TE was higher in households of higher median income (P <0.001). Prevalence of TE among those with a history of substance use was also elevated.

**Conclusion:**

Toxicologic exposure cases in the ED are elevated in particular age and race/ethnicity groups, as well as among those with a diagnosis of substance use disorder. The strength of association between these factors and TE in the general population may be different because we examined ED visits only. Further preventive and education strategies are necessary and should target the demographic groups identified in this epidemiological study.

## INTRODUCTION

Toxicologic exposures (TE) are a major preventable public health issue. Studies have shown that most exposure cases seen in the emergency department (ED) and reported to poison centers (PC) are unintentional.^1^–^5^ Vast efforts have been made in recent years to increase PC utilization as a method of reducing ED visits and decreasing unnecessary healthcare costs for low-risk exposures.^6^ The use of a PC instead of the ED has averted an estimated $16.6–$24.4 million in unnecessary healthcare costs annually in the state of Utah alone.^7^ While the use of PCs has successfully reduced the number of poisoning cases that enter healthcare facilities, there has recently been a gradual increase in the rate of health center use in certain demographic groups.^8^

Although much is known about exposure cases reported to PCs via the National Poison Data System (NPDS) published by the American Association of Poison Control Centers, there is little information regarding exposure cases from EDs.^1^ Many ED poisoning studies in the current literature have been conducted within isolated demographic groups, thus limiting their generalizability to the overall population.^2^,^4^,^9^ To develop and implement preventative strategies to decrease the rate of fatal and nonfatal TE, it is essential to identify demographic groups at higher risk. In addition, from a health policy perspective, full information on the characteristics of patients who visit the ED for TE can assist with understanding differences in population help-seeking between PCs and the more costly EDs.

In this study we sought to identify people at possible risk of TE, based on discharge information from California EDs in 2011.

## METHODS

The Agency for Healthcare Research and Quality produces the Statewide Emergency Department Database (SEDD). The Health Care Utilization Project makes available to researchers (for purchase) all visit-level data from hospitals that have an ED. Cross-validation with hospital identifiers from the American Hospital Association survey supports over 99% hospital coverage by SEDD in participating states. The SEDD contains encounter-level information on all hospital-affiliated ED visits that resulted in discharge. We obtained the most recent year of data at the time of the study with complete race/ethnicity information for the most populous state available in SEDD. Using these two criteria, we acquired California 2011 SEDD data.

We included visits identified by at least one of the following I*nternational Classification of Diseases, 9^th^ Revision* (ICD-9) codes as a TE case: E85*, and E86*. The visit was classified as substance abuse related if at least one of the following ICD-9 codes were associated with the visit: 304.0*, 304.2*, 304.3*, 304.4*, 304.7*, 305.2*, 305.5*, 305.6*, 305.7*, 965.0*, 969.7*,970,970.1, 970.8*,970.9, E850.0, E850.1, E850.2, E854.2, E854.3, E935.0, E935.1, E935.2, E939.7, E940.0, E940.1, E940.8, E940.9. We calculated the prevalence of TE in discharged ED visits per patients’ gender, race, number of chronic conditions, number of visits per year, and the median household income state quartile for patient ZIP code (MHISQ), as reported in the SEDD.

The MHISQ is a categorical variable that provides a quartile classification of the estimated median household income for each state. The cut-offs for the quartile designation are determined using ZIP code-demographic data obtained from Claritas. The assignment of MHISQ for a particular discharge is based on the median income of the patient’s ZIP code.^10^ We further categorized patients into 10-year age groups starting from 0–9 up to ≥60. Prevalence proportions are reported as cases per 100,000 patients presenting to EDs including 95% confidence intervals (CI).

**Population Health Research Capsule**

What do we already know about this issue?

*Studies have suggested that there may be racial, gender, socioeconomic, and cultural disparities that impact poison control usage, resulting in avoidable emergency department (ED) visits*

What was the research question?

*Using ED discharge data, the study aims to identify demographic groups at risk of toxicologic exposures*.

What is the major finding?

*Prevalence of exposure cases in the ED are elevated in children less than 10 years old, Caucasians, and substance users*.

How does this improve population health?

*The identification of groups at risk of toxicologic exposure can guide poison control outreach and prevention education efforts in the public health sector*.

We used SPSS Statistics 25 (IBM Corporation, Armonk, NY) for data analysis.

## RESULTS

The study included 10,124,598 ED visits in California in 2011. The prevalence of TE was 383.4 (379.6–387.3) per 100,000 visits. Table 1 shows the prevalence of TE in different patient groups.

### A. Groups with highest TE prevalence

#### A.1. Age and Gender

**Figure 1:**
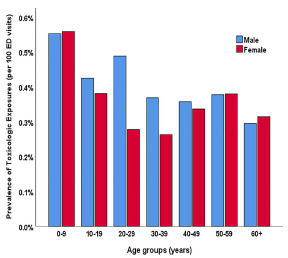
Prevalence by age group and gender of toxicologic exposure (percentages) in patients presenting to the emergency department (ED).

We found that TE was most common among patients up to age 10 (555.4, 95% CI: 544.5–566.5 per 100,000). Prevalence of TE decreased to 330.5 (325.7–335.3) per 100,000 in patients aged 30 or more. Overall, TE was more common among males. Prevalence of TE in males 20–39 years of age was 434.4 (422.7–446.3) per 100,000 in comparison with 241.3 (234.5 – 248.2) per 100,000 females of the same age group (*P* <0.001) (Figure 1).

#### A.2. Age and Gender and Race

**Figure 2:**
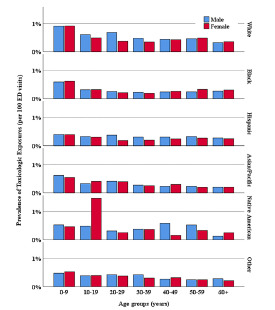
Prevalence by age group and race of toxicologic exposure cases presenting to emergency departments (ED).

Overall, White patients experienced the highest prevalence of TE compared to other racial groups (*P* <0.001). Native American female patients ages 10–19 showed a higher prevalence of TE (1,464.4, 95% CI: 802.9, 2,444.9 per 100,000) relative to all other racial groups and were the only subpopulation with a prevalence above 1,000 per 100,000 ED visits (Figure 2).

#### A.3. Age, Gender and Chronic Conditions

The prevalence of TE was elevated in people with chronic conditions. In patients aged 10–19, prevalence increased from 312.1 (300.5–324.1) per 100,000 in those with no chronic conditions, to 616.6 (582.7–651.9) per 100,000 in those with one, and 977.5 (900.1–1,059.6) per 100,000 in those with twio or more chronic conditions. Likewise, the prevalence in patients aged 20–29 increased from 268.0 (258.6–277.7) per 100,000 in those with no chronic conditions to 503.6 (481.1–526.9) per 100,000 in those with one chronic condition and 671.1 (632.7–711.1) per 100,000 in patients with two or more chronic conditions.

#### A.4. Age and Gender and Median Household Income State Quartile

**Figure 3:**
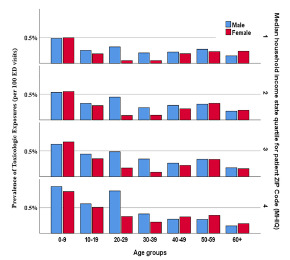
Prevalence of toxicologic exposure cases presenting to emergency departments in different age groups and median household income state quartiles per patient Zip code. *ED*, emergency department; *MHISQ*, median household income state quartile.

Prevalence of TE increased by increasing the MHISQ (*P* <0.001). In patients aged up to 39 years, the prevalence of TE rose from 358.4 (350.0–366.9) per 100, 000 in the first MHISQ to 540.0 (524.7–555.6) per 100,000 in the fourth MHISQ. Overall, the prevalence was similar in all MHISQ levels of patients above age 40 (Figure 3).

### B. Substance use

The prevalence of TE among non-substance abusers was 324.2 (320.7–327.7) per 100,000 ED visits. On the other hand, prevalence of TE among substance abusers was 4,622.9 (4,513.5–4,734.3) per 100,000 ED visits ([odds ratio (OR)]:14.90, 95% CI: 14.50–15.31).

## DISCUSSION

Toxicologic exposures remain an important public health issue in terms of lives lost and healthcare costs incurred.^9^ The use of PCs has successfully reduced the number of poisoning cases that enter healthcare facilities and has helped decrease healthcare costs.^8^ However, the use of PCs still underused, with one study reporting that 46.6% of pediatric patients who presented to the ED would have been redirected to an outpatient site had they initially called a PC.^11^ Furthermore, studies have suggested that there may be racial, gender, socioeconomic, and cultural disparities that impact PC usage.^12^ Considering the elevated healthcare costs of TE cases that present to the ED, the identification of high-risk groups in the ED is crucial for targeted exposure-prevention education. Analysis of all California ED visits for TE in 2011 that led to discharges—over 38,000 visits—highlighted several groups that are at a higher risk. These groups should be targeted for preventive measures in the public health sector. In addition, research that compares frequency of calls to PCs and visits to the ED among these subgroups may assist with targeted efforts to promote PC utilization when TE occurs.

Consistent with other studies,^1^,^13^,^14^ children ages 0–9 are at the highest risk for TE compared to other age groups. Numerous studies have attempted to understand and investigate the cause of this epidemiological finding. However, there is disagreement in the literature regarding the most common substance unintentionally ingested in children. Several studies reported that younger children ages 0–3 are more likely to be poisoned by household and non-medicinal substances.^5^,^15^,^16^ In contrast, another study reported that the rate of unintentional poisoning in children from medication was twice that of non-pharmaceutical consumer products.^4^ These differences can likely be attributed to significant variations in geographic location as well as socioeconomic and cultural factors.^14^,^16^–^18^ Previous studies also suggest that children exhibit age-related patterns with regard to the type of substance seen in TE.^15^,^19^,^20^ Furthermore, depending on their level of physical and cognitive development, young children differ widely in the severity of exposures.^21^ For children who are more physically capable of exploring their environment, it is the responsibility of the caregiver to provide greater supervision and better storage practices. Safe storage practices such as using child-resistant pill organizers and storing substances out of reach, as well as using dispensing systems, are vital in the prevention of childhood TE.^18^,^22^

A concerning finding in our results is the elevated prevalence of TE in Native American females between the ages of 10–19. Due to the limitations of our study, we could not provide a clear explanation for this finding. Future studies should analyze specific causes of TE within this subgroup.

Our results show a direct association between chronic conditions and TE in nearly all age groups. Medication errors leading to potential poisoning events are more likely to occur with chronic conditions.^23^ Studies have shown that medication errors often occur in infants/children and the elderly (>65 years old).^23^ The most common error reported is taking more than one dose at a time.^4^,^23^ Improving health literacy and numeracy skills among caretakers and patients is necessary to prevent future TE cases. Health providers should implement targeted exposure-prevention educational measures for patients with chronic diseases and their caretakers.

Our results suggest that a higher household income was associated with higher prevalence of TE in patients younger than 40 years old who presented to and were discharged from the ED. These results are inconsistent with numerous studies that have shown that TE in children occurred more frequently in lower socioeconomic groups.^5^,^6^,^13^,^24^ However, studies have suggested that higher socioeconomic status (SES) is associated with “party” drug abuse, such as γ-hydroxybutyrate (GHB), while injectable drugs are associated with lower SES.^17^,^25^ Further research can be done to analyze different potential poisoning exposures and SES.

Substance use disorder is a major health problem with significant social, mental and medical consequences. In 2011, an estimated 2.5 million ED visits resulted from medical emergencies involving drug misuse or abuse.^26^ With the recent opioid epidemic, there has been a 183% increase in opioid overdoses that present to the ED from 2004 to 2011.^27^ Unsurprisingly, our results show that a history of substance use is associated with TE in ED patients. Drug poisoning as a result of substance use often leads to serious, sometimes fatal, health consequences. Primary and secondary prevention of substance use is already a public health priority. Prevention and treatment for substance use disorder should be used to decrease occurrence of TE. Moreover, such preventive measures could significantly decrease mortality due to TE, as substance use is a well-known risk factor for morbidity, disability, and premature mortality.^9^,^28^

## LIMITATIONS

Our study has several limitations. First, the use of administrative data includes the potential for errors in recording diagnoses. Although such errors are possible, the SEDD has been widely used in numerous studies.^9^ Second, our estimates are limited to TE cases that present to the ED and led to discharges. Our data likely underestimate all cases related to TE, among them patients who may have called a PC or who presented to physician offices and were subsequently hospitalized. Further research should focus on the analysis of demographic groups at risk for more severe TE cases that resulted in hospital admission. Third, our data does not provide outcome information and is limited in its clinical utility but may be useful in primary prevention of TE.

We studied factors associated with ED presentation due to TE that resulted in discharge. The population observed in the ED does not necessarily reflect the general population, as the prevalence of medical conditions and the age of people who presented to an ED is greater than in the general population. Therefore, the associations we reported relate to help-seeking in the ED and may likely differ from prevalence in PCs and the “true” prevalence of TE as measured according to the entire state population.

## CONCLUSION

Our study identifies demographic groups at high risk of toxicologic exposure using ED discharge data. It would not be possible in a cross-sectional study to establish a causality link between patients’ characteristics and the incidence of TE. However, this does not affect the application of our findings in specifying the populations at highest priority for preventive measures. Our findings suggest increased prevalence of TE in patients who are less than 10 years old, male, and Caucasian. Our study also shows higher prevalence of TE in patients who have history of substance abuse and who have a higher median income. Further preventative and educational strategies are needed and should target the demographic groups identified in this study.

## Figures and Tables

**Table 1:** Total number and prevalence of toxicological exposure (TE) cases per 100,000 emergency department (ED) visits.

Groups	Patients in Outpatient ED	TE cases	Prevalence (per 100,000)		

			Point estimate	95% Confidence Interval	

				Lower limit	Upper limit
Age group					
0–9	1,768,544	9,823	555.4	544.5	566.5
10–19	1,127,002	4,523	401.3	389.7	413.2
20–29	1,667,254	6,026	361.4	352.4	370.7
30–39	1,320,133	4,044	306.3	297.0	315.9
40–49	1,307,667	4,507	344.7	334.7	354.9
50–59	1,152,790	4,365	378.7	367.5	390.0
≥60	1,722,942	5,272	306.0	297.8	314.4
Gender					
Male	4,527,776	19,005	419.7	413.8	425.7
Female	5,461,450	19,301	353.4	348.4	358.4
Race					
White	4,164,268	19,976	479.7	473.1	486.4
Black	1,098,837	3,366	306.3	296.1	316.8
Hispanic	3,540,937	10,834	306.0	300.2	311.8
Asian/Pacific Islander	455,081	1,439	316.2	300.1	333.0
Native American	18,588	76	408.9	322.3	511.5
Other	324,032	1,205	371.9	351.2	393.4
Number of chronic conditions					
0	5,872,776	21,118	359.6	354.8	364.5
1	2,088,472	8,462	405.2	396.6	413.9
2+	2,163,350	9,256	427.9	419.2	436.6
Number of visits per year					
1	3,373,234	12,641	374.7	368.3	381.3
2	1,655,424	5,724	345.8	336.9	354.8
3	876,909	2,965	338.1	326.1	350.5
4+	1,874,603	6,407	341.8	333.5	350.2
Median household income state quartile for patient ZIP Code					
1	3,126,047	10,867	347.6	341.1	354.2
2	2,708,825	10,150	374.7	367.5	382.1
3	2,308,298	9,060	392.5	384.5	400.7
4	1,713,820	7,612	444.2	434.3	454.2
